# Stemness and EMT profiles shift in xenografts derived from cisplatin-sensitive and cisplatin-tolerant ovarian cancer cells

**DOI:** 10.1371/journal.pone.0342326

**Published:** 2026-02-05

**Authors:** Alena Mrkvicova, Marcela Slavickova, Eva Peterova, Lucie Melounkova, Helena Parova, Radim Havelek, Anna Krejcova, Renata Kohlerova, Jana Nekvindova, Petra Kazimirova, Milena Hajzlerova, Tomáš Rozkoš, Martina Rezacova

**Affiliations:** 1 Department of Medical Biochemistry, Faculty of Medicine in Hradec Kralove, Charles University, Hradec Kralove, Czechia; 2 Department of Clinical Biochemistry and Diagnostics, University Hospital Hradec Kralove, Hradec Kralove, Czechia; 3 Department of Environmental and Chemical Engineering, Faculty of Chemical Technology, University of Pardubice, Czechia; 4 The Fingerland Department of Pathology, University Hospital Hradec Kralove, Hradec Kralove, Czechia; Universite du Quebec a Trois-Rivieres, CANADA

## Abstract

The transcriptional alterations underlying epithelial-to-mesenchymal transition (EMT) in chemoresistant ovarian cancer remain a matter of debate, with emerging evidence pointing to tumour cell plasticity and subclone reprogramming. In this study, we developed a cisplatin-tolerant ovarian cancer model by treating the cisplatin-sensitive OVCAR-3 cell line with a single dose of cisplatin, generating the OVCAR-3 CP variant. These cisplatin-tolerant cells exhibited distinct EMT-related changes at both transcriptomic and protein levels, potentially regulated by epigenetic mechanisms. EMT profiling revealed that OVCAR-3 CP cells did not display a pronounced mesenchymal phenotype but rather retained epithelial characteristics and showed elevated expression of *ALDH3A1*. In contrast, the parental chemosensitive OVCAR-3 cells expressed canonical mesenchymal markers (*CDH2, VIM, ZEB1/2, SNAIL, SLUG*) and lacked stemness marker expression. Upon xenografting, both OVCAR-3 and OVCAR-3 CP cells demonstrated phenotypic plasticity, with parental OVCAR-3 xenografts acquiring EMT-like features resembling to those observed in cisplatin-tolerant tumours. These findings suggest a decoupling of EMT from cisplatin-tolerance and instead underscore a stronger association between stemness traits and cisplatin tolerance. Our data further indicate that xenografting can induce significant cellular reprogramming. Comprehensive characterization of ovarian cancer cell-derived xenograft is therefore essential, as they represent a valuable translational platform for investigating therapy adapted ovarian cancer cells.

## Introduction

Chemoresistance remains a significant challenge in the treatment of ovarian cancer, often leading to treatment failure and disease progression. Cancer cells exposed to chemotherapy can acquire the ability to survive and proliferate despite drug-induced stress through a range of adaptive mechanisms. These include enhanced DNA repair pathways, expression of transporter proteins reducing the intracellular concentration of drugs, activation of metabolic pathways reducing drug toxicity, improving sell-renewal capabilities and cooperation with tumour microenvironment that provide a protective and supportive environment [1–4]. Understanding the mechanisms underlying CP resistance is crucial for developing effective therapeutic strategies and improving patient outcomes.

Importantly, tumour cells do not respond uniformly to chemotherapy. Drug exposure can select from a heterogeneous tumour cells population, giving rise to clones with transient or stable drug tolerance. These clones may exhibit self-renewal capacity, slow cycling or senescent behaviour or sustained proliferative resistance. This heterogeneity has started the debate as to whether drug-tolerant persisters (DTPs), dormant tumour cells (DTCs), cancer stem cells (CSCs) represent distinct cell populations or instead reflect a spectrum of stress-adapted tumour cells [5]. Interestingly a single-cell study show that chemotherapy selects for multiple resistant cell fates that differ in growth behaviour and gene expression and are often influenced by cellular states already present before treatment [6].

An intriguing connection has been observed between chemotherapy tolerance and the epithelial-to-mesenchymal transition (EMT), a cellular program where epithelial cells undergo morphological changes to adopt a mesenchymal phenotype. The transcriptional and epigenetic alterations underlying EMT, such as deregulation of cadherin expression and the activation of transcription factors Snail, Slug and Zeb have been associated with chemoresistance [7–10]. EMT can drive chemoresistance by enhancing drug efflux, inducing cell cycle arrest, and promoting an anti-apoptotic state [11]. On the other hand, the pressure exerted by chemotherapeutic drugs on tumour cells can in turn stimulate expression of EMT-related transcription factors as an adaptive survival mechanism. Cisplatin was reported to promote EMT features in osteosarcoma cells *in vitro* [12] and induced EMT migration and metastasis in lung adenocarcinoma *in vivo* [13]. In the recurrent head and neck squamous cell carcinoma proliferating Slug-positive tumour cells were observed and expand after therapy [14]. Others reported cisplatin as an effective treatment against EMT [15]. These contradictory findings suggest that EMT related responses to chemotherapy are context-dependent and heterogeneous, potentially reflecting the coexistence of multiple drug-adaptive cell states within the same tumour.

Cancer xenograft research is a type of preclinical cancer research where human cancer cells (cell derived xenografts, CDX) or patient cancer cells (patient derived xenografts, PDX) are transplanted into immunodeficient mice to study cancer cell phenotype plasticity, tumour growth progression and the treatment response. Although PDXs models for ovarian carcinoma have been shown to maintain histopathologic and genetic stability [16] differences in transcriptional signatures related stromal cells were reported [17] indicating the need for further investigation.

In this study, we generated a cisplatin-selected ovarian cancer cell population by exposing the cisplatin-sensitive OVCAR-3 cell line to a single dose of cisplatin, yielding the OVCAR-3 CP variant. This selection resulted in a sustained reduction in cisplatin sensitivity, stable proliferative capacity *in vitro* and *in vivo*, and marked alterations in EMT-associated phenotypes. Importantly, these cells maintained reduced cisplatin sensitivity in the absence of continued drug exposure, indicating a therapy adapted state rather than transient drug tolerance. Moreover, these transformed cells showed different tumour-forming behaviours in immunodeficient mice and displayed unique EMT phenotype influenced by epigenetic changes. By comprehensively analysing these cellular and molecular characteristics, we aimed to gain insights into the mechanisms driving CP tolerance and identify potential therapeutic targets for overcoming resistance in ovarian cancer.

## Results

### Generation and characterisation of CP-tolerant OVCAR-3 CP cells

In this study, we developed a CP-tolerant OVCAR-3 cell line, termed OVCAR-3 CP, from the chemosensitive parental OVCAR-3 cells through single-dose CP treatment. The OVCAR-3 cell line is a high-grade serous ovarian adenocarcinoma model originally established from ascitic fluid of a patient clinically refractory to cisplatin. Despite this fact OVCAR-3 cells display sensitivity to CP [18]. Initially, OVCAR-3 cells were exposed to 1 μM CP for 48 hours, leading to apparent cellular damage characterized by vacuolization and necrosis. Following a 4-day recovery period, the cells were passaged. Proliferating cell clusters observed in subsequent days were isolated, manually dissociated, and replanted. A representative phase‑contrast images illustrating the workflow used to generate the cisplatin‑selected OVCAR‑3 CP subline are provided (S1 Fig, panel A). OVCAR-3 CP cells were expanded for several passages, cryopreserved, and later thawed and propagated without cisplatin to confirm stability of the cisplatin-tolerant phenotype prior to downstream analyses.

To assess epithelial junctional organization following cisplatin selection, we examined the expression and subcellular localization of the tight-junction protein ZO-1 by immunofluorescence microscopy (S1 Fig, panel B). In parental OVCAR-3 cells, ZO-1 was prominently localized at cell-to-cell junctions, showing continuous membrane-associated staining. In contrast, cisplatin-selected OVCAR-3 CP cells retained detectable ZO-1 expression but showed a less uniform and more diffuse staining pattern. This suggests disruption of cell-to-cell junctions, a feature previously associated with increased invasive properties and linked to intracellular signalling changes related to EMT [19].

**Fig 1:**
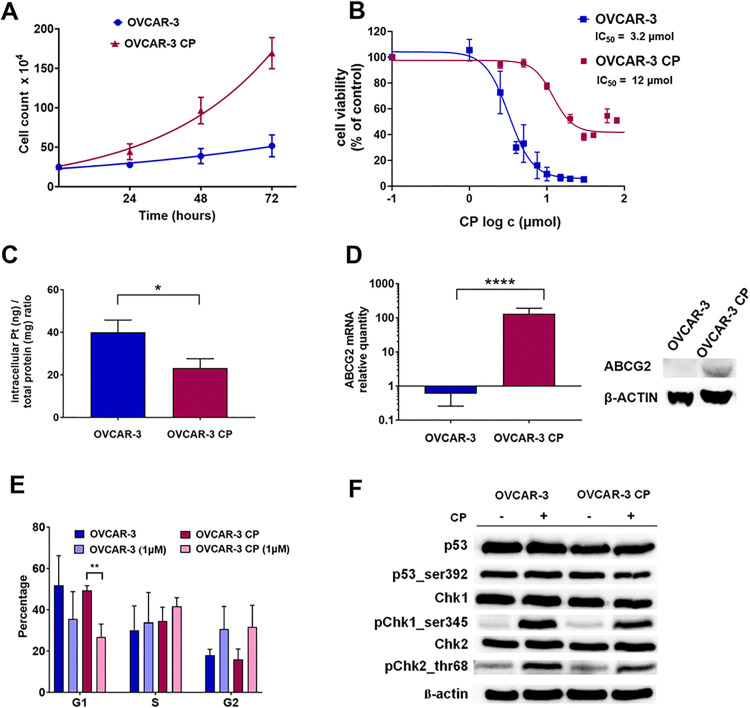
Characterization of OVCAR and OVCAR-CP cells. (A) The doubling time was determined using a proliferation curve derived from manual cell counting. (B) Cell proliferation and viability were quantified via the colorimetric WST-1 assay. Data are shown as the percentage relative to untreated control cells, which were arbitrarily set at 100%. Each data point represents the mean ± standard deviation (SD) from four independent experiments. (C) Intracellular cisplatin (CP) concentrations were measured using inductively coupled plasma mass spectrometry (ICP-MS) in parental OVCAR-3 and CP-tolerant OVCAR-3 CP cells. Results are expressed as the ratio of platinum (Pt) mass (ng) to one mg of total cellular protein. Each point is the mean ± SD from three independent experiments. (D) ABCG2 gene expression levels were quantified by qPCR. Data are shown as means ± SD from three independent experiments. ABCG2 protein expression was assessed by Western blot analysis. (E) The distribution of OVCAR-3 and OVCAR-3 CP cells across the G1, S, and G2/M phases of the cell cycle was analysed by flow cytometry. The bar graph presents quantitative data on cell cycle distribution, with percentages expressed as mean ± SD from three independent experiments. (F) Protein expression levels of p53 and its phosphorylated form at serine 392, along with checkpoint kinases Chk1 phosphorylated at serine 345, and Chk2 phosphorylated at threonine 68, were detected by Western blot analysis. The representative blots shown were selected from at least three independent experiments, unprocessed blots are provided in S1_raw_images.pdf. β-actin was used as a loading control. Statistical significance was assessed using Student’s t-test with *P ≤ 0.05, **P ≤ 0.01, and ****P ≤ 0.0001 indicating increasing levels of significance.

We first examined the proliferation rates of both OVCAR-3 and OVCAR-3 CP cell lines. As shown in Fig 1A, the OVCAR-3 CP cells exhibited a higher proliferation rate compared to the parental OVCAR-3 cells, with doubling times of 26 h for OVCAR-3 CP and 62 h for OVCAR-3, respectively. To assess the CP tolerance of the newly established OVCAR-3 CP cells, we performed the WST-1 assay. Cellular sensitivity to CP was determined for both the parental and CP-tolerant cell lines, with dose-response curve and IC_50_ values presented in Fig 1B. The IC_50_ value for the OVCAR-3 CP cells was nearly four times higher than that of the parental line (IC_50_ OVCAR-3 = 3.2 μM, IC_50_ OVCAR-3 CP = 12.0 μM).

To determine whether the reduced CP sensitivity was associated with changes in intracellular concentrations of CP, we quantified the intracellular platinum (Pt) levels using ICP-MS. There was a significant disparity in intracellular Pt concentrations between the two cell lines as depicted in Fig 1C. Specifically, the parental OVCAR-3 cells accumulated 39.9 ± 3.3 ng Pt per mg of total cellular protein, whereas the CP-tolerant OVCAR-3 CP cells contained 23.2 ± 2.5 ng CP per mg of protein, indicating a 42% reduction in intracellular Pt accumulation in the OVCAR-3 CP cells compared to the parental OVCAR-3 cell line.

It is well-established that ABC transporters are instrumental in reducing drug accumulation in multidrug-resistant cells. We measured ABCG2 mRNA expression using qPCR in both cell lines, revealing a significant 219-fold upregulation in the OVCAR-3 CP cells compared to the parental OVCAR-3 cells (Fig 1D), which was further confirmed at the protein level by Western blot analysis.

Flow cytometric analysis was conducted to investigate the distribution across cell cycle phases in both cell lines. As depicted in Fig 1E, a 24 h treatment with 1 μM CP negatively affected the proportion of cells residing in the G1 phase of the cell cycle. A notable reduction in the G1 phase cell proportion was observed, decreasing from 51.8 ± 8.2% to 35.6 ± 7.6% in OVCAR-3 cells and from 49.5 ± 1.2% to 26.7 ± 3.7% in OVCAR-3 CP cells. Additionally, a modest increase in the G2 phase fraction was observed, although it was statistically non-significant: from 18.1 ± 1.6% to 30.6 ± 6.3% in OVCAR-3 cells and from 16.0 ± 2.9% to 31.6 ± 6.1% in OVCAR-3 CP cells. Upon exposure to CP OVCAR-3 showed mild differences in cell cycle distribution when compared to OVCAR-3 CP.

To further explore the molecular responses induced by CP treatment, we utilized Western blotting to analyse proteins involved in the DNA damage response. As depicted in Fig 1F, both cell lines expressed P53 and its phosphorylated variant on Ser392, with no noticeable changes post-CP exposure. Additionally, the protein levels of checkpoint kinases Chk1 and Chk2 were comparable in untreated OVCAR-3 and OVCAR-3 CP cells. Following CP treatment (48 h), we found an upregulation in the phosphorylation levels of Chk1 (at serine 345) and Chk2 (at threonine 68) in both cell variants.

### Characterization of the tumour xenograft derived from OVCAR-3 and OVCAR-3 CP cells

**Fig 2:**
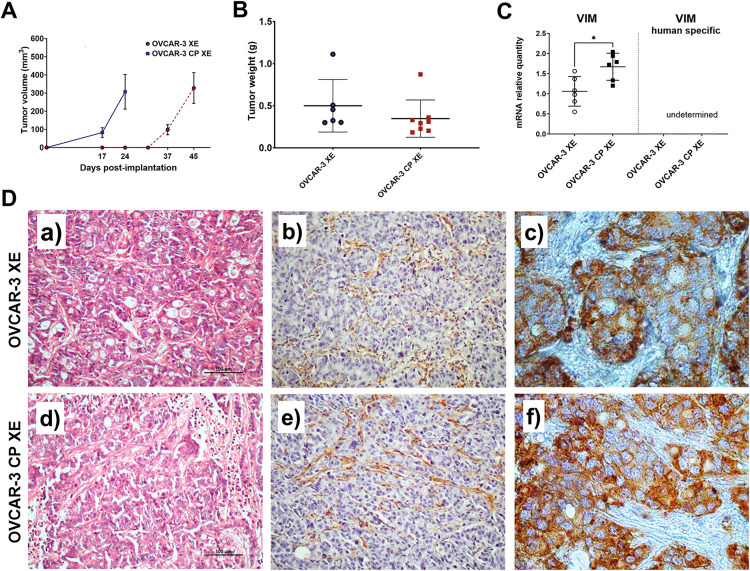
Characterization of CDX derived from OVCAR-3 and OVCAR-3 CP cell. A line graph depicts the growth of tumour volume (mean ± SEM) over time (in days post-implantation) for both OVCAR-3 XE and OVCAR-3 CP XE xenografts (panel A). A scatter plot shows the final tumour weight in grams (panel B). Each point represents the mean ± SD (with tumours measured bilaterally in each animal).. A comparison of vimentin mRNA expression is presented, distinguishing between human-derived and mouse-derived vimentin transcripts in the tumour tissue of the OVCAR-3 XE and OVCAR-3 CP XE xenografts (panel C). Panel D shows histopathological characteristics of the xenograft tissue. Haematoxylin and eosin (H&E)-stained sections of tumours from both OVCAR-3 XE and OVCAR-3 CP XE are shown in images (a) and (d), respectively. Additionally, immunostaining for vimentin is shown in images (b) and (e), and for HLA class I in images (c) and (f). Positive staining is indicated by brown coloration, while nuclei are counterstained with haematoxylin. The original magnification is 200× for H&E sections and 600× for anti-vimentin and anti-HLA-stained sections.

To investigate ability of OVCAR-3 and OVCAR-3 CP cells to develop tumours *in vivo* we established CDX model using BALB/c-nude mice. Mice were subcutaneously inoculated with the OVCAR-3 and OVCAR-3 CP cancer cells. Tumour size was measured weekly, and tumour volumes were calculated. Tumour growth kinetics of xenografts derived from the OVCAR-3 XE and OVCAR-3 CP XE cell lines are summarized in Fig 2A. After implantation, OVCAR-3 CP XE tumours (n = 8 tumours from four mice; bilateral; blue squares) exhibited a brief latency phase: volumes were still small at day 17, but then increased rapidly, reaching 307 ± 95 mm^3^ by day 24. In contrast, parental OVCAR-3 XE tumours (n = 6 tumours from three mice; bilateral; red circles) displayed a prolonged latency, with volumes remaining undetectable until day 31. Only after day 37 did these tumours enter a rapid expansion phase, growing to 327 ± 84 mm^3^ by day 45 (Fig 2A). Despite differences in latency and growth kinetics, both tumour types ultimately reached comparable tumour volumes, (Fig 2B).

We performed immunohistochemical (IHC) staining on tumour sections derived from these two xenograft models, OVCAR‐3 XE and OVCAR‐3 CP XE (Fig 2D). Sections were stained with haematoxylin‐eosin (panels a and d), anti‐vimentin (panels b and e), and anti‐HLA class I ABC antibodies (panels c and f). Tumour samples from both models exhibited common features, such as nuclear atypia and pleomorphism. Morphologically, the tumours resembled high‐grade serous adenocarcinoma, displaying predominantly solid and solid‐cribriform patterns, with a minority of areas showing tubular or microcapillary formation. Foci of necrosis were also evident, reflecting aggressive tumour growth.

Notably, each tumour nodule was surrounded by connective‐tissue septa rich in spindle shaped fibroblast like cells, which we suspected to be of murine origin. To test this, we performed immunohistochemistry using an antibody against human HLA class I. Tumour cells stained strongly for HLA (brown), whereas the fibroblast‐like cells within the septa were HLA‐negative, confirming their murine derivation. In both xenograft models, these septa contained vimentin-positive cells consistent with fibroblasts. To quantify stromal vimentin expression, we carried out qPCR for *VIM* transcripts (Fig 2C). No human-specific VIM mRNA was detected in any tumour sample (Fig 2C, left panel, human-specific *VIM*), indicating that all *VIM* expression originated from the murine stromal compartment. This was confirmed by mRNA quantification using *VIM* assays designed to detect both human and cross-reactive mouse *VIM* transcripts. Moreover, OVCAR-3 CP XE xenografts showed significantly higher levels of murine *VIM* mRNA than the OVCAR-3 CP.

### EMT marker characterization of tumour xenograft and their parental cell

**Fig 3:**
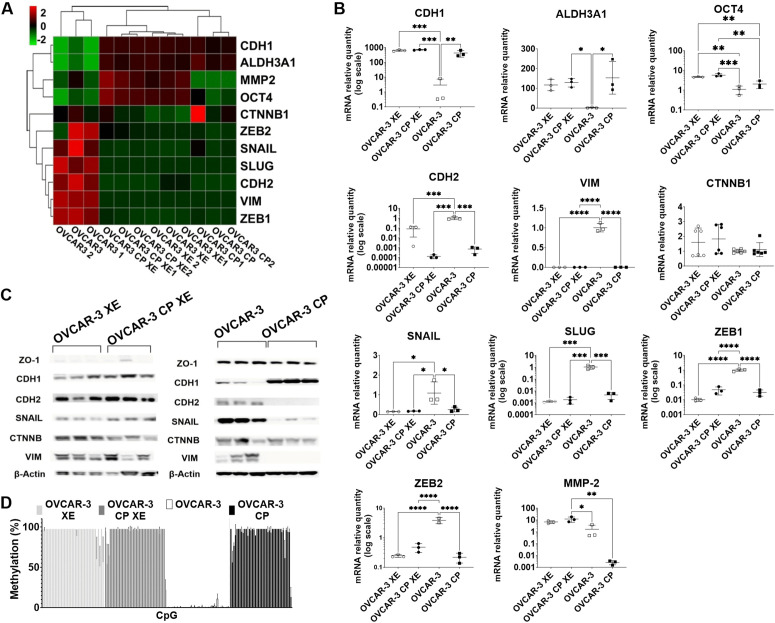
Characterization of the OVCAR-3 and OVCAR-3 CP tumour xenografts. Panel A shows a heatmap of the log_2_ transformed gene expression profiles across OVCAR-3, OVCAR-3 CP, OVCAR-3 XE and OVCAR-3 CP XE samples (n = 3). Each row represents an individual gene, while each column corresponds to a sample. The colour scale ranges from green (indicating downregulation) to red (indicating upregulation). Panel B displays individual plots of mRNA expressions of CDH1, CHD2, CTNNB1, ALDH3A, SOX4, ZEB1, ZEB2, SNAIL, SLUG and MMP2 measured using qPCR. Graphs show the individual values and mean and SD of the three independent experiments. Significance was tested using one-way ANOVA, followed by Dunnet’s post-hoc test (*P ≤ 0.05; ** P ≤ 0.01; ***P ≤ 0.001; **** P ≤ 0.0001). Panel C represents western blot analysis of EMT markers (ZO-1, CDH1, CDH2, SNAIL, CTNNB, VIM, and β-actin as a loading control). The representative blots shown were selected from at least three independent experiments, unprocessed blots are provided in S1_raw_images.pdf. Panel D shows bar chart of CHD2 methylation changes in individual CpG sites in cell lines and OVCAR-3 XE and OVCAR-3 CP XE xenograft tissue. Analysis was performed in three independent experiments. Means ± SD are shown (n = 3).

We investigated the expression of a range of EMT markers in both chemosensitive OVCAR-3 and CP-tolerant OVCAR-3 CP ovarian cancer cell lines, along with their corresponding xenograft tumors (Fig 3). The chemosensitive OVCAR-3 cells exhibited a distinct expression profile, whereas the CP-tolerant OVCAR-3 CP cells and both xenograft models (OVCAR-3 XE and OVCAR-3 CP XE) shared similar EMT characteristics (Fig 3A) as illustrated through a heatmap depicting log_2_ transformed mRNA expression levels of these EMT markers.

Individual gene expression plots are shown in Fig 3B, with relative mRNA levels for each gene normalized to those in parental OVCAR-3 cells. We observed a significant elevation in the mRNA expression of the epithelial marker *CDH1*, which increased by 142-fold. In contrast, mesenchymal-related genes such as *CDH2* and *VIM* demonstrated substantial downregulation by factors of 1428-fold and 500-fold, respectively, when compared to parental OVCAR-3 cells. Moreover, transcription factors linked to the EMT process including *SNAIL, SLUG, ZEB1, and ZEB2,* were markedly reduced in OVCAR-3 CP cells (fold changes of 4, 217, 33, and 18 correspondingly). Additionally, the expression of gelatinase A (*MMP-2*) was decreased by 800-fold; conversely, the pluripotency marker *ALDH3A1* was significantly upregulated by an equal factor in OVCAR-3 CP cells.

OVCAR-3 XE tumours exhibited striking change in EMT markers expression compared to OVCAR-3 cells cultured *in vitro*, indicating a partial reversion to an epithelial-like phenotype post-implantation: OVCAR-3 XE tumours showed lower *CDH2, VIM, SNAIL, SLUG, ZEB1,* and *ZEB2* levels and higher *CDH1* and *OCT4* levels compared to the original OVCAR-3 cells. In contrast, OVCAR-3 CP XE did not show significant phenotypic changes to *in vitro* cultured OVCAR-3 CP cells, retaining EMT characteristics except for a 4600-fold increase in *MMP-2*. It is also interesting that no significant differences in EMT markers were found between OVCAR-3 XE and OVCAR-3 CP XE xenografts, suggesting similar molecular profiles.

We assessed protein levels using Western blot analysis for EMT markers ZO-1, CDH1, CDH2, SNAIL, CTNNB1, and VIM (Fig 3C). The results revealed differences in EMT marker expression between the two cell lines, with OVCAR-3 CP cells showing increased CDH1 and decreased CDH2, SNAIL, and VIM levels. No major differences were found in xenograft tissues, indicating consistent protein levels across xenograft tumours. Since our anti-vimentin antibody can detect both human-and mouse-derived proteins, we previously assessed non-human vimentin presence within tumour tissue.

The results underscore critical alterations related to EMT that transpired following cisplatin selection among ovarian cancer cells while also illustrating how this phenotype may be reversible post-cell implantation into murine models at both mRNA and protein levels.

Finally, we examined potential epigenetic regulation of *CDH2* expression by performing next-generation sequencing (NGS)-based methylation analysis of the *CDH2* promoter/exon region (Fig 2D). The CP-tolerant OVCAR-3 CP cell line exhibits pronounced hypermethylation at CpG islands within the *CDH2* promoter/exon region – methylation levels were nearly 100% at all CpG sites, which is associated with a significant reduction in *CDH2* mRNA expression. In contrast, the parental OVCAR-3 cell line displays a distinct methylation profile at this locus, indicating an epigenetic divergence between the two cell lines.

Upon xenografting, this distinction is no longer preserved. Xenografts derived from both OVCAR-3 CP cells (OVCAR-3 CP XE) and parental OVCAR-3 cells (OVCAR-3 XE) demonstrated nearly complete methylation (approaching 100%) at all CpG sites within the *CDH2* promoter/exon region. Notably, the methylation pattern observed in OVCAR-3 XE xenografts closely mirrored that of the OVCAR-3 CP XE tumours. These findings suggest that xenografting induces epigenetic changes in the parental OVCAR-3 cells, resulting in a methylation profile resembling that of the CP-tolerant phenotype.

### Correlation of dysregulated EMT genes in tumour xenografts derived from sensitive and CP-tolerant parental cells

**Fig 4:**
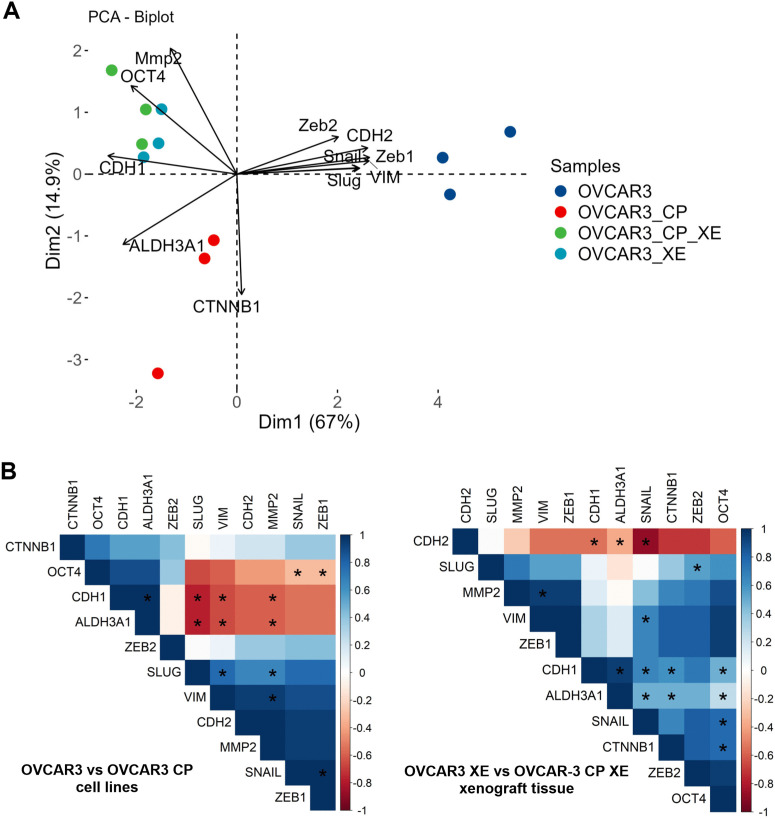
PCA analysis and correlation of EMT genes. Panel A displays the biplot a result of a PCA analysis performed on the mRNA expression profiles across OVCAR-3, OVCAR-3 CP, OVCAR-3 XE and OVCAR-3 CP XE samples. Each point represents an individual sample, plotted according to its coordinates on the two dimensions (Dim1 and Dim2) that capture the greatest variance. Vectors indicate how strongly each variable contributes to each principal component, with the direction and length of each vector reflecting its correlation with PC1 and PC2. Samples clustering together share similar profiles, while those that separate along the axes exhibit distinct characteristics. Panel B shows a corelation heatmap illustrating Spearman’s correlation of the EMT-related gene expression in OVCAR-3 and OVCAR-3 CP (left panel) and in OVCAR-3 XE and OVCAR-3 CP XE (right panel). Positive correlations are shown in blue, whereas negative correlations are shown in red. Colour intensity reflects the strength of the correlation. Asterisks (*) denote statistically significant correlations with p < 0.05.

To further examine the global expression patterns of epithelial-mesenchymal transition (EMT) markers across four experimental groups, specifically OVCAR-3, OVCAR-3 CP, and their corresponding xenografts, we performed an unsupervised principal component analysis (PCA). The resulting two-dimensional score plots illustrated the EMT expression profiles for each sample, with vector arrows highlighting the influence of individual EMT markers on the principal components. The PCA biplot (Fig 4A) revealed a significant relationship among mesenchymal markers (*CDH2, SNAIL, SLUG, VIM, ZEB1, ZEB2*, and *MMP-2*) present in OVCAR-3 cells. This gene cluster effectively distinguished OVCAR-3 cells from both OVCAR-3 CP and the associated xenograft tissues. Additionally, the PCA biplot confirmed that the EMT expression profiles of the xenografts OVCAR‐3 XE and OVCAR‐3 CP XE were similar, yet distinctly separated from the cell line samples.

The correlation plot (Fig 4B) demonstrated a positive correlation between *CDH1* and the stemness marker *ALDH3A1* in both OVCAR-3 and OVCAR-3 CP cells. In contrast, a negative correlation was observed between *CDH1* and both *SLUG* and *VIM*, as well as with *ALDH3A1*. Furthermore, *OCT* exhibited a negative correlation with *SNAIL* and *ZEB1*. The correlation heatmap of EMT markers within the tumour xenografts revealed a distinctive pattern: a strong positive correlation was identified between *OCT4* and *CTNNB* with *SNAIL*, while *CDH2* displayed an inverse relationship with *SNAIL*. Additionally, a negative correlation was noted between *CDH1* and *CDH2*.

These findings indicate that not only do mRNA expression patterns change upon implantation into mice, but the correlations among these markers also vary when tumour xenografts are established.

## Discussion

Understanding the molecular aspects of chemoresistance is crucial for developing efective strategies that can help to overcome treatment failure. Increasing evidence indicates that drug-tolerant persister cells survive chemotherapy and act as a source of tumour regrowth, often exhibiting stemness-associated traits, partial mesenchymal features, and enhanced antioxidant defense [20–22]. Although EMT is well recognized for its role in cancer progression and metastasis, its contribution to chemotherapy tolerance and resistance remains debated. In this study, we developed and characterized a stable cisplatin (CP)-tolerant OVCAR-3 cell line through a single-dose selection process without the need for additional drug exposure. By utilizing CDX tumours derived from both the sensitive OVCAR-3 and CP-tolerant OVCAR-3 CP cell lines, we evaluated of molecular alterations associated with induced chemoresistance *in vivo*.

Initially, we characterized the CP-tolerant OVCAR-3 CP cell line, noting changes in drug uptake mechanisms, ABC transporter expression, and DNA damage response pathways. In our model, selecting the sensitive OVCAR-3 cells with a single dose of CP led to the propagation of a CP-tolerant clone, OVCAR-3 CP. Compared to the sensitive cells, our CP-tolerant clone exhibited a reduced doubling time and demonstrated a four-fold decrease in CP sensitivity, as well as lower CP accumulation and upregulation of ABCG2. Limited CP accumulation could be mechanistically attributed to the upregulation of the drug transporters, which expels CP from the cancer cells. Upregulated expression of *ABCG2* in OVCAR-3 cells in response to CP treatment was also observed by others [23]. CP resistance of various tumour cell types often involves multiple cellular changes, including variations in the influx or efflux of the drug across the cell membrane [24,25], alterations in the drug’s detoxification processes via platinum-GSH conjugates [26,27], modifications in the DNA damage response, suppression of programmed cell death, and the upregulation of proteins that prevent apoptosis [28].

Our data support the emergence of cisplatin-adapted tolerant cells. This concept builds on previous descriptions of reversible, chromatin- and epigenetically mediated drug-tolerant states that arise following therapy, in which rare pre-existing transcriptional variability determines which cells survive initial treatment. Therapeutic stress can subsequently drive epigenetic reprogramming, stabilizing these adaptive states and promoting more persistent tolerant or resistant behavior. Such plastic and reprogrammable intermediate states have been proposed to occupy a continuum between reversible drug tolerance and irreversible resistance [21,22]. These findings reinforce the view that early tolerance mechanisms can be biologically meaningful and may contribute to tumour persistence and relapse after chemotherapy.

These chemotherapy-selection not only enable OVCAR-3 CP cells to evade cisplatin-induced cell death but also enhance their capacity to initiate, establish, and grow as xenograft tumours. In chemotherapy-naïve epithelial ovarian cancer (EOC) samples, successful engraftment was associated with increased tumorigenicity, a more aggressive disease phenotype, and poorer patient prognosis [29]. However, this pattern was not observed in CP-tolerant cells, suggesting that their ability to form xenografts may be influenced by distinct mechanisms unrelated to initial tumour aggressiveness. The PDX models of EOC did not alter drug sensitivity, supporting their use for testing chemotherapy responses *in vivo*. Moreover, PDXs that responded well to treatment were linked to better patient outcomes [30].

In this study, we established CDX models using both CP-sensitive and CP-tolerantOVCAR-3 and OVCAR-3 CP cells, evaluating consistency in phenotype and EMT status. The resistant CDX formed tumours in half the time compared to the sensitive ones, indicating greater tumorigenicity. Despite this difference, both CDX types showed similar histological features. The tumour masses were enriched with murine stromal cells, including fibroblast-like cells expressing vimentin, a marker of cancer-associated fibroblasts (CAFs). Vimentin⁺ CAFs are linked to tumour growth, metastasis, recurrence, drug resistance, and poor prognosis across several cancers. Vimentin⁺ CAFs presence may indicate higher tumour malignancy and is significantly associated with increased disease severity [31]. Additionally, other studies have shown that, following xenografting, host cells organize into a supportive murine stroma that includes endothelial cells and cancer-associated fibroblasts [17,32,33]. The interactions between tumour cells and host stroma may recapitulate key aspects of tumour stroma observed in patients, yet they are frequently overlooked. CAFs are known to be critical components of the TME, as they remodel the extracellular matrix, secrete growth factors and cytokines, and modulate immune cell responses; these functions contribute to tumour progression and therapeutic resistance. Patient PDX models demonstrates that in vivo environments reshape tumour cell populations, selecting for different subclones and phenotypes [34]. Incorporating host stromal interactions, particularly CAF dynamics, into preclinical models is therefore essential for accurately recapitulating patient tumour microenvironments and improving treatment strategies.

EMT is a well-known mechanism that contributes to chemoresistance in cancer cells. During this process, epithelial cells lose cell-to-cell adhesion and acquire a more migratory, mesenchymal-like phenotype. Recent discussions on how to define EMT have emphasized the need to assess multiple types of markers such as transcription factors, cytoskeletal proteins, and membrane-associated proteins. These discussions also highlight that EMT is a bidirectional process where cells exist on a phenotypical spectrum from epithelial to mesenchymal states, with various subpopulations of cells displaying distinct phenotypes [35]. Our study provides insights into these dynamics by comparing EMT patterns between cisplatin-sensitive OVCAR-3 cells and their CP-tolerant counterpart, OVCAR-3 CP, in both *in vitro* and *in vivo* settings. We identified an EMT shift toward more epithelial characteristics in the CP-tolerant OVCAR-3 CP cells. Specifically, while the parental OVCAR-3 cells exhibited high expression of mesenchymal markers (e.g., *CDH2* and *VIM*) and transcription factors (e.g., *ZEB1*, *ZEB2*, *SNAIL*, and *SLUG*), the CP-tolerant OVCAR-3 CP cells and the xenografts (OVCAR-3 CP XE and OVCAR-3 XE) displayed a contrasting profile. OVCAR-3 cells lacked expression of stemness markers such as ALDH3A1 and OCT-4, whereas the CP-tolerant OVCAR-3 CP cells expressed these markers significantly. Xenografts derived from CP-tolerant cells also showed elevated expression of these markers. Additionally, upon implantation, OVCAR-3 cells acquired an EMT signature similar to CP-tolerant tumours.

Previous studies have shown that chemoresistant ovarian cancer cells, such as OV-90 and SKOV-3, exhibit downregulation of CDH1 and upregulation of CDH2 and VIM [36]. Similarly, increased expression of VIM and Slug was observed in the OVCA 433 cell line following cisplatin treatment [37]. Resistant ovarian cancer A2780cis cells also showed upregulation of EMT-related transcription factors, including Snail, Slug, Twist2, and ZEB2 [38]. However, another study reported a contrasting finding, where epithelial-like ovarian cancer cells were found to be more resistant to cisplatin [39]. These discrepancies may reflect the unique characteristics of different cell lines, including their phenotype, response patterns, and the specific chemotherapy type, dose, or duration involved. This highlights the potential need for personalized treatment strategies in ovarian cancer, tailored to the EMT status of the tumour cells.

The concept of cancer cell plasticity supports the existence of a intrinsically tolerant subpopulation, such as CSC within the tumour, capable of surviving chemotherapy - CSCs. CSC are known to facilitate tumour regrowth even after a substantial portion of the tumour has been destroyed [40]. Platinum tolerant cells (persister or CSCs) exhibit specific features such as expression of ABC drug transporters, high ALDH enzyme activity, that reduces oxidative stress and detoxifies anticancer agents and thus promoting resistance [41]. In ovarian cancer, for example, ALDH-positive CSCs are highly chemoresistant and tumorigenic [42]. CSCs also maintain low intracellular ROS levels through enhanced antioxidant pathways and metabolic reprogramming. Furthermore, CSCs display cellular plasticity and are capable of undergoing EMT, leading to increased invasiveness, metastasis, and therapy resistance [43]. Together, these observations support an emerging model in which EMT, stemness, and drug tolerance are interconnected but not identical programs. While EMT has been associated with chemoresistance in many cancer types, growing evidence emphasizes epithelial-mesenchymal plasticity rather than complete transition as a key determinant of therapy adaptation. In this context, hybrid states with enhanced self-renewal capacity may be particularly effective at surviving cytotoxic stress without requiring full EMT transition.

One of the most striking findings of this study is the convergence of EMT-related phenotypes between parental OVCAR-3 and OVCAR-3 CP cells following xenografting. In vivo, parental OVCAR-3 tumours acquired EMT-like features resembling those observed in cisplatin-selected cells, despite their chemosensitive origin in vitro. This phenotypic convergence highlights the profound influence of the tumour microenvironment on cellular state and underscores the limitations of extrapolating in vitro phenotypes directly to in vivo behaviour.

These findings suggest that EMT-like programs observed in xenografts may reflect microenvironment-driven reprogramming rather than intrinsic chemoresistance. Stromal interactions, hypoxia, extracellular matrix remodelling, and paracrine signalling from fibroblast-like cells are all known to promote EMT-associated transcriptional programs and may override baseline epithelial or mesenchymal identities. Thus, EMT observed in vivo should be interpreted as a context-dependent adaptive response rather than a fixed marker of drug resistance.

Several limitations of the present study should be acknowledged. The cisplatin-selected OVCAR-3 CP cells exhibit a moderate shift in cisplatin sensitivity rather than high-level, genetically fixed resistance. While this limits extrapolation to advanced platinum-resistant disease, it also reflects a relevant therapy-adapted state, which is increasingly recognized as critical for tumour persistence and relapse. This study focused on a single ovarian cancer cell line model. While OVCAR-3 provides a well-characterized and clinically relevant system for investigating epithelial-mesenchymal plasticity and therapy adaptation, comparing these findings to additional ovarian cell lines, will be necessary. Use of bulk mRNA and protein analyses limits resolution of intra-population heterogeneity. Emerging evidence indicates that drug tolerance, EMT states, and stemness-associated traits may coexist within complex cellular mixtures. Single-cell transcriptomic and lineage-tracing approaches would therefore be required to delineate the relationships among these adaptive states and to identify rare subpopulations driving tolerance and tumour regrowth. Finally, although stromal fibroblast-like cells were observed and characterized within xenografts, comprehensive characterization of CAF subtypes and their functional contributions to cisplatin tolerance was beyond the scope of this study. Co-culture models will be important to fully understand microenvironment-driven resistance mechanisms.

## Conclusion

Bridging the gap between drug sensitivity and resistance will require an integrated research approach that combines mechanistic insights from single-cell-based models with studies capturing the complexity of tumour responses to chemotherapy. In this context, EMT, stemness, and drug tolerance should be viewed as interconnected but distinct adaptive programs. Tumour cell populations comprise a heterogeneous portfolio of stress-adapted states, including drug-tolerant, dormant, stem-like, and hybrid phenotypes, that can interconvert depending on treatment context, duration, and microenvironmental cues rather than existing as fixed, discrete entities.

## Materials and methods

### Cell culture and treatment

The human ovarian cancer cell line OVCAR-3 was obtained from the American Type Culture Collection (ATCC) (VA, USA) and cultured in RPMI-1640 Medium (Gibco®, Thermo Fisher Scientific, U.S.) supplemented with 20% foetal bovine serum (FBS) (Biowest, Miami, USA), and 100 U/mL penicillin (Sigma-Aldrich, St. Louis, USA). Cells were maintained at 37°C in a 5% CO_2_ atmosphere.

### Induction of cisplatin tolerance

CP-tolerant OVCAR-3 CP variants were derived from the original parental OVCAR-3 cell line following a single exposure to cis-Diammineplatinum(II) dichloride (CP) (Sigma-Aldrich, St. Louis, USA) at 2.5 µM. Initially, OVCAR-3 cells were treated with cisplatin for 48 hours. Subsequently, the medium was removed, and cells were allowed to recover for four days, followed by cultivation for one week. The cells exhibited sensitivity to the treatment, resulting in significant damage. CP-tolerant cells were selected and further cultivated, achieving stable CP-tolerant after six weeks of growth without cisplatin. Stock solutions were freshly prepared by dissolving 10 mg of CP in 10 mL of cultivation medium to achieve a 3.33 mM concentration, then diluted to 1 µM for subsequent experiments.

### Measurement of cell proliferation

Cell proliferation was assessed using the WST-1 assay (Roche, Mannheim, Germany). Cells were seeded at 5 x 10^3^ cells/well in 100 µL culture medium. After 24 hours, cells were treated with CP at concentrations ranging from 2.5 to 320 μM for OVCAR-3 CP and 1–50 μM for OVCAR-3. After an additional 48 hours, 50 µL of WST-1 reagent was added and the absorbance was measured at 440 nm with a reference wavelength of 690 nm after 150 minutes of incubation using multiple plate readers (Tecan Infinite M200, Switzerland). Experiments were performed in triplicate to calculate the IC50 values for each cell line.

### Determination of doubling time

OVCAR-3 and OVCAR-3 CP cells were seeded at 2.5 x 10^5^ cells per Petri dish in 2 mL of culture medium. Cell viability was assessed every 24 hours for four days using a Bürker chamber with Trypan Blue staining (Sigma-Aldrich, St. Louis, USA). Doubling time was calculated from three independent measurements using GraphPad Software (San Diego, California, USA).

### Cell cycle distribution

The parental OVCAR-3 cell line, and its CP-tolerant variant (OVCAR-3 CP) were treated with 1 μM CP for 24 h. Untreated cells served as the control group. Following treatment, cells from each group were harvested by trypsinization, washed with ice-cold Dulbecco’s phosphate-buffered saline (DPBS), fixed in 70% ethanol, and stored at 4 °C for subsequent cell cycle distribution analysis. Prior to staining, fixed cells were centrifuged to remove ethanol and rinsed twice with ice-cold DPBS. To assess low molecular-weight DNA fragmentation, cells were incubated for 5 minutes at room temperature in a phosphate–citrate buffer (192 mL of 0.2 M Na_2_HPO_4_ and 8 mL of 0.1 M citric acid, pH 7.8). After an additional wash with ice-cold DPBS, cells were stained with propidium iodide in Vindelov’s solution and incubated for 1 h at 37 °C. DNA content was measured using a Beckman Coulter CytoFLEX LX flow cytometer (Beckman Coulter, Miami, FL, USA) with excitation at 488 nm and emission detection between 600–620 nm. Data analysis was performed using Kaluza Analysis 2.1 software.

### Measurement of intracellular Pt content using ICP MS

Determination of platinum (the isotope ^195^Pt) was carried out using ICP-oTOF-MS spectrometer Optimass 8500 (GBC Scientific Equipment, Australia) equipped with the concentric nebuliser MicroMist (400 μL.min^-1^) coupled to the thermostated (10 °C) cyclonic spray chamber (both Glass expansion, Australia). The operating conditions of the ICP-MS analysis were: the sample flow rate 600 μL.min^-1^; plasma power 1250 W; plasma, auxiliary and nebuliser gas flow rates were 13, 0.55 and 0.92 L.min^-1^, respectively, and multiplier gain 2650, multiplier gain 2650 V, acquisition time 5 s, three replicates. Working isotopes about possible isobaric overlaps of interfering ions with the same mass have been selected. The external aqueous calibration (standard solutions 0.01-0.05-0.1-0.5-1-5 μg. l^-1^) was used with the internal standard ^205^Tl. The limits of detection (the concentration equal to three times the standard deviation of intensity near the isotope mass) were 0.4 ng. l^-1^.

### RNA isolation and qPCR analysis

Cells were washed with PBS, harvested, and lysed in lysis buffer. Total cellular RNA was extracted using the RNeasy Mini Kit according to the manufacturer’s instructions (Qiagen, Hilden, Germany). One µg of RNA was reverse transcribed using a cDNA Reverse Transcription Kit and quantified with TaqMan Gene Expression Assays as described elsewhere [16]. The relative expression was measured using the Quantstudio 6 Real-Time PCR system (Applied Biosystems, CA, USA). mRNA levels were calculated using the comparative Ct method.

### Bisulfite next generation sequencing

Illumina platform with targeted amplicon sequencing approach was employed to determine methylation levels of selected CpG sites in the promoter/exon 1 regions of CDH1 and CDH2 genes. Genomic DNA from cell lines was extracted using the QIAmp DNA Mini Kit (Qiagen, Hilden, Germany) and then bisulfite-converted using EZ DNA Methylation-Gold™ Kit according to the manufacturer’s protocol (Zymo Research Corporation, Irvine, CA, USA). Specific primers for amplification were designed in the on-line methylation primer designing software MethPrimer [44]. Amplicon information and primer sequences with added adapters are listed in S1 Table.

Sequencing libraries were prepared using the Multiplicom MID for Illumina MiSeqTM kit (Agilent Technologies, Santa Clara, CA, USA). Next-generation sequencing was performed on the MiSeq System (Illumina, San Diego, CA, USA) using Reagent Kit v2 with 2x250 bp paired-end reads following the manufacturer’s instructions. Data were analysed using NextGENe® software version 2.3.4.5 (Softgenetics, State College, PA, USA). Bisulfite-converted methylated DNA was used as a reference sequence. All experiments were carried out in triplicates.

### Western blot

Cells were harvested and suspended in PBS containing 4 mM EDTA, then washed three times. Proteins were extracted using Cell Lysis Buffer (Cell Signalling Technology, Danvers, USA). Protein content was determined using the Bicinchoninic Acid Protein Assay Kit (Sigma-Aldrich, St. Louis, USA). Fifty µg of protein were subjected to SDS-PAGE and Western blot with immunochemical detection as described elsewhere. The EMT-related primary and secondary antibodies were included in the Epithelial-Mesenchymal Transition Antibody Sampler Kit obtained from Cell Signalling Technology (Danvers, MA, USA). Staining with primary monoclonal mouse anti-β-actin (Sigma-Aldrich, St. Louis, USA) was used as a loading control. The goat anti-mouse secondary antibody and the detection system Western Blotting Luminol Reagent were purchased from Santa Cruz Biotechnology (Dallas, USA). The blots were scanned using the PXi imaging system (Syngene, Cambridge, UK).

### In vivo tumour xenograft models

This study was approved by the Charles University, Faculty of Medicine in Hradec Kralove Ethical Committee, and the MSMT, project number 12981/2019–4 and the experiments were carried out in accordance with Czech Law No. 246/1992 Sb. Researchers and animal-facility staff were adequately trained in surgical techniques and animal handling in accordance with Czech Law No. 246/1992 Sb.

Six-week-old female BALB/c-nude mice were housed in an environmentally controlled room (22 ± 2°C, 40–60% humidity, and a 12 h light cycle). OVCAR-3 and OVCAR-3 CP cells (3 × 10^6^ in 300 µL PBS) were subcutaneously inoculated into both sides of the backs of the mice (n = 4, per group). The animals’ well-being was monitored daily, no anaesthesia was required. Tumour size was measured weekly, and volumes were calculated using the formula 0.5 × (width^2^ × length). Tumours developed in three mice injected with OVCAR-3 cells and in four mice injected with OVCAR-3 CP cells. When tumour volumes reached approximately 300 mm^3^ the mice were euthanized. Animals were euthanized under deep general anaesthesia induced by intramuscular ketamine (100 mg/kg) and xylazine (5 mg/kg). Euthanasia was completed by cervical dislocation as a secondary physical method to ensure death. No mouse deaths were observed during the experiment.

After sacrifice, tumour masses derived from OVCAR-3 cells (n = 6) and from OVCAR-3 CP cells (n = 8) were excised, weighed, and either fixed in 10% neutral buffered formalin for histological analysis or preserved at −70 °C for methylation and Western blot studies or in RNA later solution for qPCR analysis.

### Immunofluorescence

Cells grown on plastic chamberslides (Sigma-Aldrich, St. Louis, USA) were fixed in 4% paraformaldehyde at room temperature for 10 min. Permeabilized for 15 min with 0.2% Triton X-100, and blocked with 1% FBS for 30 min. The cells were incubated with appropriate primary antibody. The ZO-1 (D7D12) rabbit monoclonal antibody included in Epithelial-Mesenchymal Transition Antibody Sampler Kit was obtained from Cell Signalling Technology (Danvers, MA, USA). The secondary antibody was FITC-conjugated AffiniPure Donkey Anti Rabbit IgG (Jackson Immunoresearch Europe, Newmarket, UK); it was diluted 1:200 and the cells were incubated in the dark for 1 hour. Nuclei were stained with 4′,6-diamidino-2-phenylindole (DAPI, Sigma-Aldrich). Unspecific mouse immunoglobulins served as negative controls. Cells were washed with PBS, mounted in Vectashield (Vector Laboratories, Burlingame, California) and studied under Nikon Eclipse fluorescent microscope.

### Histological and immunohistological analysis

Tumour samples were fixed in 10% buffered formalin for several days at 4°C and embedded in paraffin. Five-micron sections were stained with haematoxylin and eosin. Histological sections were examined microscopically and analysed quantitatively using ImageJ software. For immunohistochemical staining, rabbit monoclonal antibodies anti-Vimentin (D21H3) (cat. nr. 5741) Cell Signalling Technology (Danvers, MA, USA), and Anti-HLA Class 1 ABC antibody (EMR8−5) (cat. Nr. ab70328 Abcam, Cambridge, UK), were used at dilutions of 1:200 and 1:1000, respectively. The LSAB plus system-HRP served as the secondary antibody. Samples were stained with DAB Chromogen (Dako, Glostrup, Denmark) and counterstained with haematoxylin.

### Statistical analysis

Results are presented as mean ± SD. Student’s t-test was employed to compare two groups of normally distributed interval data. One-way ANOVA and Kruskal-Wallis tests were used to compare three or more groups for normally and non-normally distributed interval data, respectively. Calculations were performed using GraphPad Prism version 6.00 for Windows (GraphPad Software, San Diego, California, USA). Multivariate data analysis was performed using FactoMineR and Factoextra R package [45,46] in the RStudio 2023.06.1.

## Supporting information

S1 Table Amplicon characteristics and primer sequences of CDH2. Amplicon characteristics and primer sequences used in NGS methylation analysis of the CDH2 promoter/exon region.
(XLSX)

S1 Fig Generation of cisplatin‑selected OVCAR‑3 CP cells and ZO‑1 localization. (A) Representative phase‑contrast images illustrating the workflow used to generate the cisplatin‑tolerant OVCAR‑3 CP subline. Parental OVCAR‑3 cells were exposed to 1 μM cisplatin for 48 h, resulting in extensive cellular damage. After a 4‑day recovery period and first passage, surviving cell clusters were isolated, and expanded to establish the OVCAR‑3 CP population. (B) Immunofluorescence staining for the tight‑junction marker ZO‑1 (green) with nuclear counterstain DAPI (blue) in parental OVCAR‑3 and OVCAR‑3 CP cells. Original magnification 600x.
(TIF)

S1_raw_images Raw images from Western blot analysis of selected proteins. Uncropped and unprocessed blots are shown. The blots are representative of independent biological replicates. β-actin was used as a loading control. Molecular weights and exposure times are indicated. Red “X” marks indicate lanes or bands not used in the final figures.
(PDF)
