# Supplementary material for: Stemness and EMT profiles shift in xenografts derived from cisplatin-sensitive and cisplatin-tolerant ovarian cancer cells
Source: PLoS One. 2026 Feb 5;21(2):e0342326. doi: 10.1371/journal.pone.0342326 (PMC12875467; doi:10.1371/journal.pone.0342326)

Fig. 1 panel D—publication

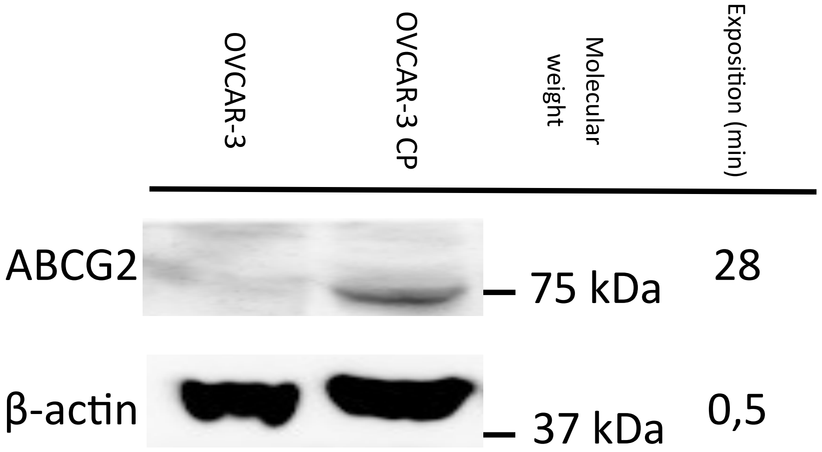

Fig. 1 panel D—other blotts

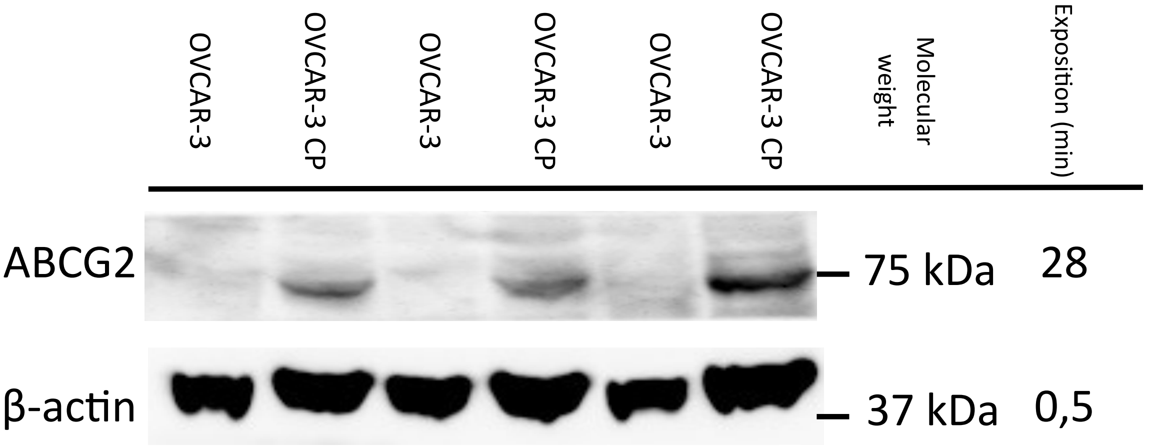

Fig. 1 panel F—publication

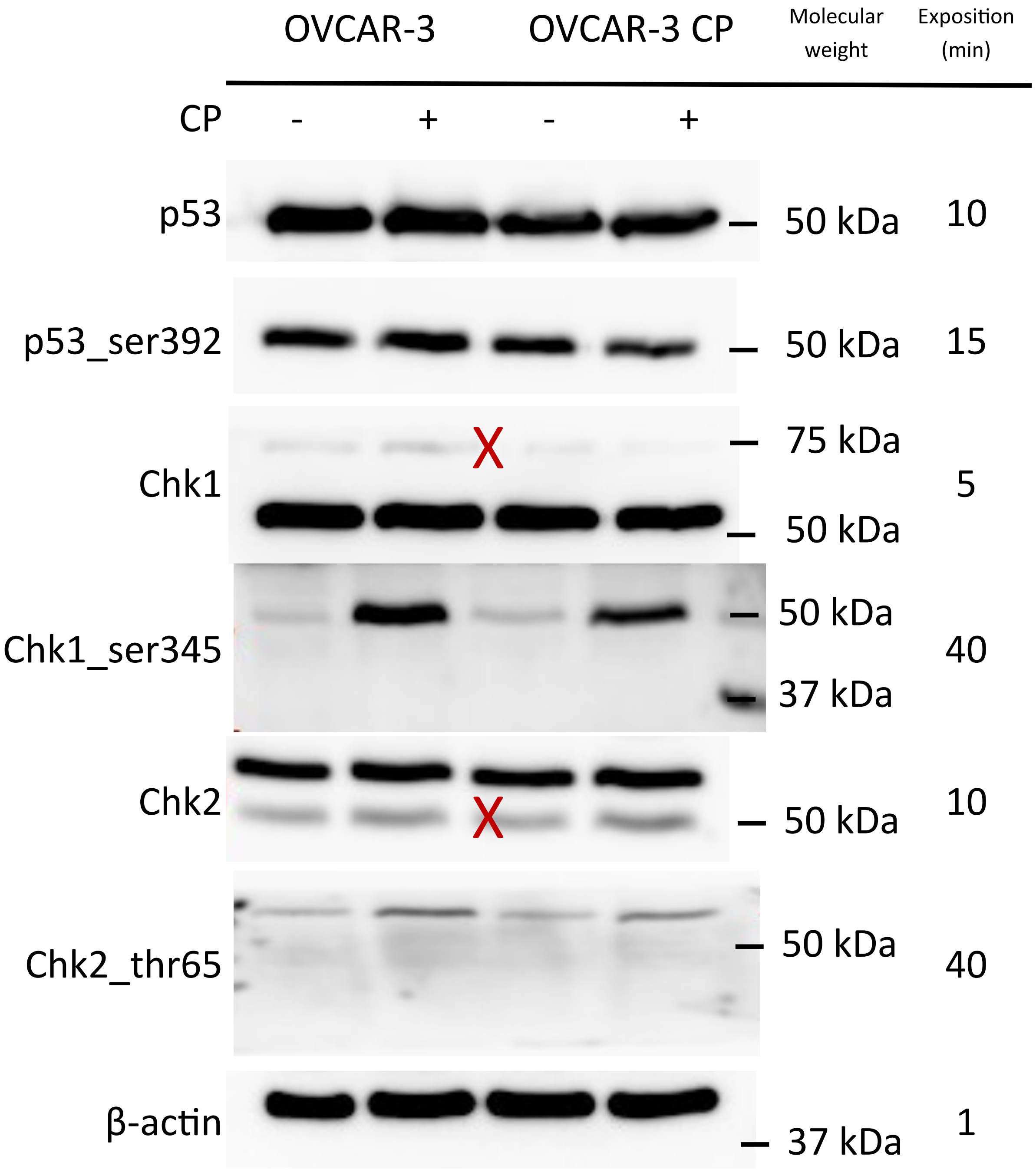

Fig. 1 panel F—other blotts

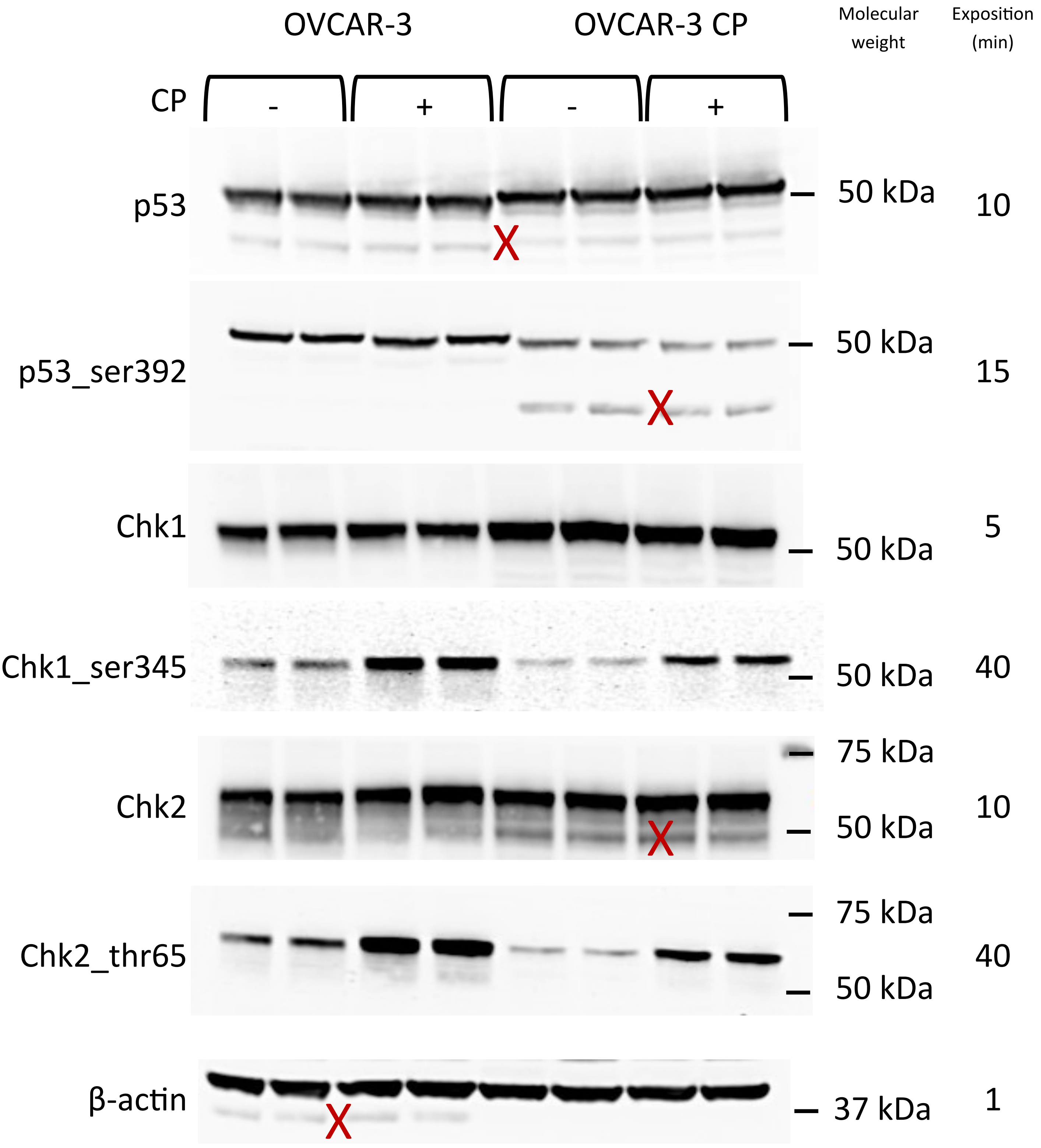

Fig. 3 panel B—publication

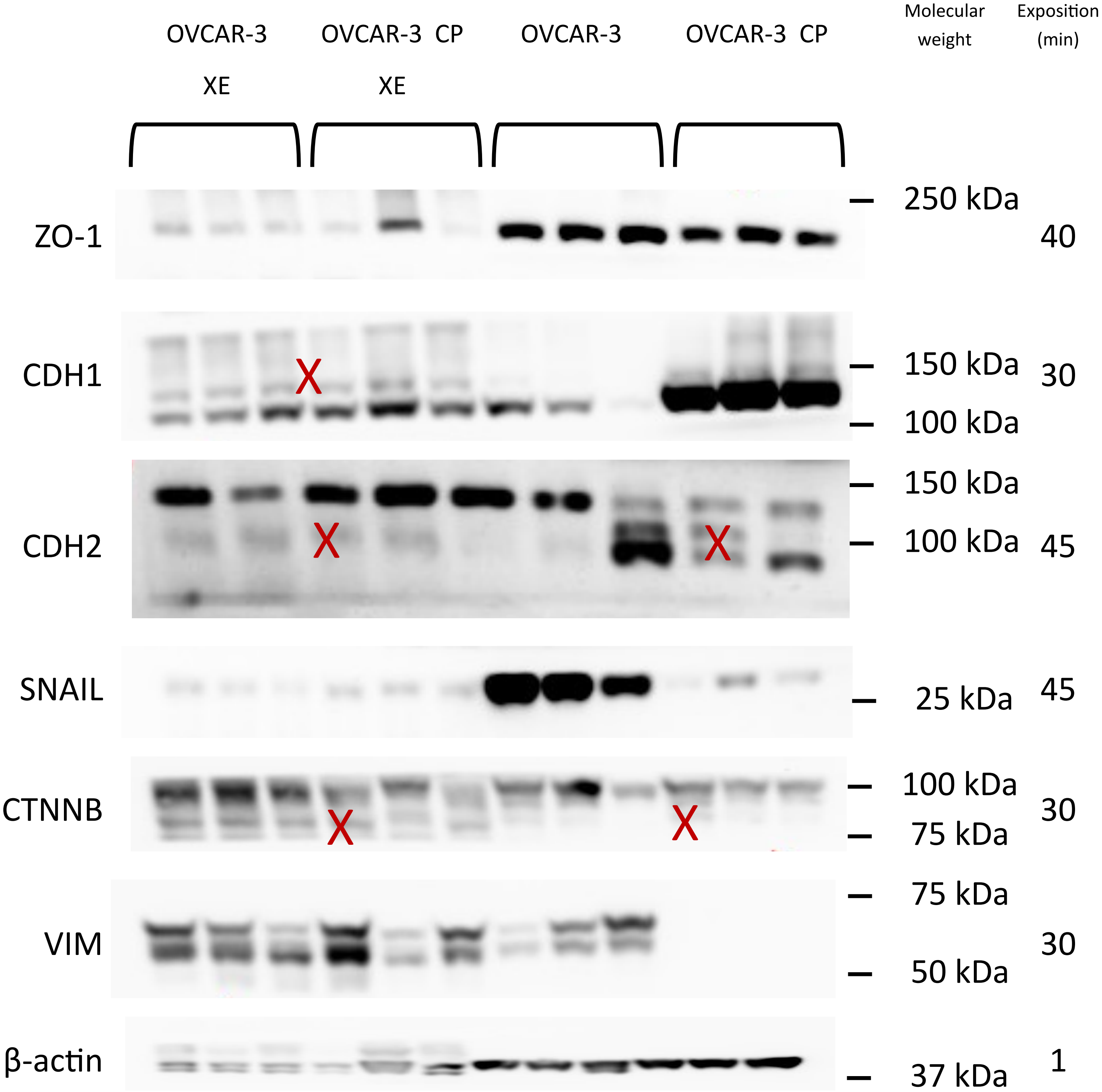

Supplement: S1_raw_images — Uncropped and unprocessed blots are shown. The blots are representative of independent biological replicates. β-actin was used as a loading control. Molecular weights and exposure times are indicated. Red “X” marks indicate lanes or bands not used in the final figures. (PDF) [file pone.0342326.s003.pdf]
